# Hybrid electromagnetic toroidal vortices

**DOI:** 10.1126/sciadv.ads4797

**Published:** 2025-02-21

**Authors:** Ren Wang, Beier Ying, Shuai Shi, Junsong Wang, Bing-Zhong Wang, Musheng Liang, Yijie Shen

**Affiliations:** ^1^ Institute of Applied Physics, University of Electronic Science and Technology of China, Chengdu 611731, China.; ^2^ Yangtze Delta Region Institute (Huzhou), University of Electronic Science and Technology of China, Huzhou 313098, China.; ^3^ Centre for Disruptive Photonic Technologies, School of Physical and Mathematical Sciences, Nanyang Technological University, Singapore 637371, Singapore.; ^4^ School of Electrical and Electronic Engineering, Nanyang Technological University, Singapore 639798, Singapore.

## Abstract

The ubiquitous occurrence of toroidal vortices, or vortex rings, in fluid dynamics scenarios in nature has garnered notable attention in the scientific community, while their electromagnetic counterparts have only been proposed recently with two distinct manifestations: vector toroidal pulses and scalar phase toroidal vortices. Herein, we theoretically propose a form of electromagnetic toroidal vortex solutions that uniquely integrates both scalar and vector characteristics, challenging the prevailing notion of their mutual exclusivity. We also present an experimental generation of hybrid toroidal vortex pulses by a compact coaxial horn emitter augmented with a radial metasurface. These topological pulses exhibit peculiar electromagnetic features, such as vortex streets, skyrmions, and transverse orbital angular momentum, and may present advantages when propagating through perturbations, opening avenues for enhanced free-space information transmission, topologically nontrivial light-matter interaction, and microscopy techniques.

## Teaser

Toroidal electromagnetic pulses are upgraded to host vortex streets, skyrmion topology, and transverse orbital angular momentum.

## INTRODUCTION

The phenomenon of toroidal vortices, also known as vortex rings, is widely observed in nature (*1*). These vortices manifest in various forms such as bubble rings, smoke rings, and mushroom clouds, and have been seen in the locomotion of flagellates (*2*), spore dispersal (*3*), dandelion flight (*4*), cumulus clouds (*5*), drop splashing (*6*), and blood flow through the heart (*7*). The distinctive and intriguing topological quasiparticle structure of toroidal vortices has led to extensive research across multiple scientific fields. These vortices have been identified or intentionally created in diverse environments, including tokamaks (*8*), nuclei (*9*), heterogeneous media (*10*), Bose-Einstein condensates (*11*), pseudospin structures (*12*), magnetic bubbles (*13*), droplet manipulation (*14*), and shock-accelerated interfaces (*15*). In addition to isolated toroidal vortices, the interactions between different toroidal vortices have garnered notable scholarly interest (*16*–*18*).

**Fig. 1.**
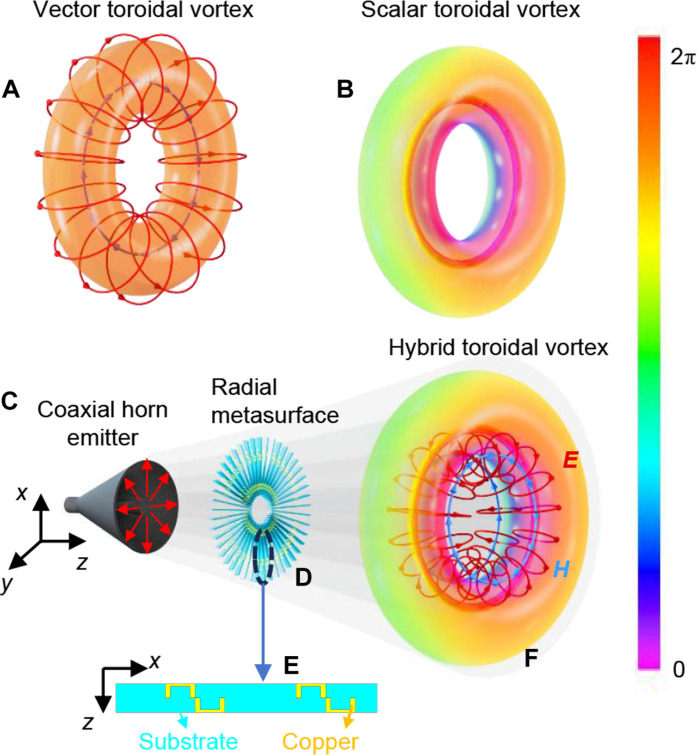
Scheme for generating HETVs. (**A**) Vector and (**B**) scalar toroidal vortices. (**C**) Coaxial horn emitter with inner and outer conductors, enabling the generation of radially polarized pulses. (**D**) Radial metasurface with (**E**) radially arranged metasurface subarrays, each composed of two metasurface units. The radial metasurface converts radially polarized waves into scalar toroidal vortices, inducing a phase variation of 2π, represented by colors, around the surface of a spatiotemporal torus along a poloidal coordinate. The singularities of the scalar toroidal vortex induce saddle points, resulting in the generation of vector electromagnetic toroidal vortices, delineating a toroidal surface with electric field lines. The vector and scalar toroidal vortices exhibit coupled topologies and spatial relationships, forming a type of electromagnetic pulse: (**F**) HETV.

In the realm of electromagnetics, scholars have proposed two kinds of independent electromagnetic toroidal vortices: vector and scalar (*19*). Vector electromagnetic toroidal vortices, denoted as field-line toroidal pulses (*20*), are characterized by an array of neighboring electric or magnetic field lines, delineating a toroidal surface at a distance from the optical axis, as shown in Fig. 1A. They exhibit skyrmion topology (*21*–*23*), have anapole-excitation capability (*24*), and have been used for three-dimensional (3D) superresolution localization (*25*). In contrast, scalar electromagnetic toroidal vortices, referred to as phase-vortex toroidal pulses (*26*), induce a phase variation of 2π around the surface of a torus along a poloidal coordinate, as shown in Fig. 1B. This results in the formation of an optical vortex with torus structure, representing a form of spatiotemporal vortices with transverse orbital angular momentum (OAM) (*27*–*31*). Recently, vector electromagnetic toroidal vortices have been observed through methodologies involving metasurfaces (*20*), quantum control (*32*), coaxial horn antennas (*33*), or gold nanotorus (*34*), whereas scalar electromagnetic toroidal vortices have been observed using techniques like conformal transformations or symmetry-breaking gratings (*26*, *35*, *36*).

In this paper, we proposed an additional family of electromagnetic toroidal pulses—hybrid electromagnetic toroidal vortices (HETVs)—and present a method for generating such pulses using coaxial horn antennas equipped with radial metasurface. HETVs exhibit peculiar electromagnetic features, such as vortex streets, skyrmions, and transverse OAM.

## RESULTS

### Generation scheme for HETVs

The device for generating HETV in an electromagnetic pulse primarily consists of a coaxial horn emitter and a specially designed radial metasurface, as shown in Fig. 1. The coaxial horn emitter, which features inner and outer conductors (Fig. 1C), has been proposed for generating electromagnetic vector toroidal pulses (*33*). The coaxial horn emitter can emit radially polarized electromagnetic waves, characterized by a radial polarization null point and a longitudinal polarization peak along the propagation axis (*33*). To transform the radial polarization component into waves containing scalar toroidal vortices, we positioned a purposely designed radial metasurface in front of the coaxial horn emitter (Fig. 1D). Considering an arbitrary radial range, the transfer function of radial metasurface subarray can be expressed as (*37*)

$$H\left(k_{r},\omega \right)\approx S_{r}k_{r}/k_{0}+S_{t}\left(\omega -\omega_{0}\right)/\omega_{0}$$ (1)

where *k_r_* is the transverse wave vector along the radial direction, ω is the angular frequency of plane wave centered at ω_0_ with a wave vector *k*_0_, *S_r_* and *S_t_* are two complex constants determining amplitude and phase distribution in the *k_r_* − ω domain. The overall metasurface is coaxial with the emitter and composed of radially arranged metasurface subarrays (Fig. 1E), each subarray containing two asymmetric metasurface units, each made of a substrate and two oppositely oriented C-shaped copper foils. Detailed structures and dimensions of these units are provided in the Supplementary Materials (note S2). Spatiotemporal vortex pulses can be generated based on the breaking of spatial mirror symmetry (*30*, *31*). The two oppositely oriented C-shaped metallic slabs in the metasurface unit create an asymmetric structure, which produces a phase distribution of e^−ilθ^ in the spatial frequency-frequency domain. Therefore, *S_r_* and *S_t_* can be normalized to 1 and a π/2 (OAM mode: *l* = −1) or −π/2 (OAM mode: *l* = +1) phase difference is required between *S_r_* and *S_t_*. A radially polarized output field $E_{r}\left(r,t\right)$ can be

$$E_{r}\left(r,t\right)=s\left(r\right)h\left(r,t\right)\text{exp}\left(-i\omega_{0}t+{\mathrm{ik}}_{0}z\right)$$ (2)

where s(r) is the radially polarized component of incident wave and h(r,t) is the inverse Fourier transform of the transfer function of radial metasurface subarray. The longitudinal components $E_{z}$ can be determined by applying Gauss’s law in free space, followed by numerical integration (*20*)

$$E_{z}\left(r,z\right)=-\int_{\alpha }^{z}\frac{E_{r}\left(r,z\prime \right)}{r}+\frac{\partial E_{r}\left(r,z\prime \right)}{\partial r}\mathrm{dz}$$ (3)

where α is selected as a reference point where the field is zero. Combining the longitudinally polarized component $E_{z}$ with the transversely polarized component $E_{r}$ enables the construction of the spatiotemporal vector field distribution, where the singularities of the scalar toroidal vortex and radial polarization induce saddle points to form the vector toroidal vortices. In topologically electromagnetic fields, such as toroidal vortices or skyrmions, saddle points occur at specific locations where the field distribution changes from a locally converging to a locally diverging behavior. The inner saddle points of the vector toroidal vortices generated by this method are located along the propagation axis, while the outer saddle points are at the phase singularities of the scalar toroidal vortices. Thus, the vector and scalar toroidal vortices exhibit coupled topologies and spatial relationships, forming a type of electromagnetic pulse: HETV, as shown in Fig. 1F, whose scalar toroidal vortex phase distribution is mapped according to the given color bar. Within a few half-wavelengths around the vortex singularity in the HETV, several vector toroidal vortices connect, forming an electromagnetic vortex street and several skyrmion textures, as detailed in the following results. See the Supplementary Materials (note S1) for detailed derivation of the HETV.

**Fig. 2.**
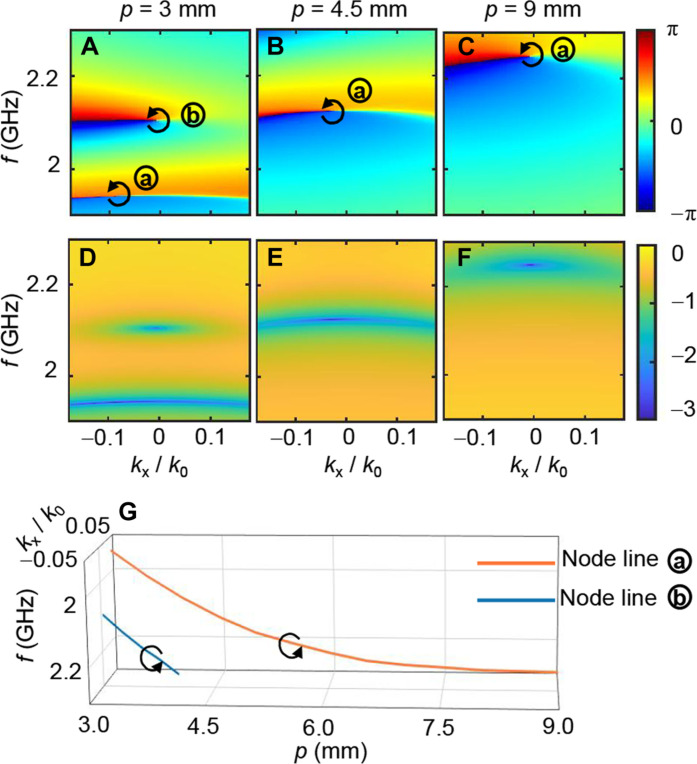
Transmission spectrum function of asymmetric metasurface unit. (**A** to **C**) Phase response and (**D** to **F**) amplitude response. (**G**) Singularities for different *p* values. When the subarray arrangement period, *p*, varies between 2.4 and 7.7 (corresponding to the period of unit simulated under Floquet boundary condition along the *y* axis, also roughly corresponding to the distance between adjacent metasurface subarrays in the radial metasurface), there is always one vortex phase singularity with the same handedness in the 2 to 2.2 GHz range. This ensures that when incident pulses are in the 2 to 2.2 GHz range, the radial metasurfaces can convert radially polarized electromagnetic waves into spatiotemporal vortices at each radial position, thereby forming a scalar toroidal vortex.

Our asymmetric metasurface unit is designed with a periodic boundary, as detailed in the Supplementary Materials (note S2). As shown in Fig. 1D, the distance between neighbored radial metasurface subarrays varies along the radius, which necessitates the designed asymmetric metasurface unit to have periodic robustness. The asymmetric modulation of the incident pulse can be illustrated by the transmission spectrum function $T\left(k_{x},\omega \right)$, where ω is the angular frequency of a plane wave, and $k_{x}$ is the wave vector component along the asymmetric metasurface unit. When varying the spacing period *p* (corresponding to the period of unit simulated under Floquet boundary condition and also roughly corresponding to the distance between adjacent metasurface subarrays in the radial metasurface), the phase and amplitude responses of the designed unit’s transmission spectrum function are shown in Fig. 2 (A to C and D to F, respectively). As the period *p* increases, the vortex phase singularities shift gradually to higher frequencies. The positions of the singularities for different values of *p* are shown in Fig. 2G. When *p* varies between 2.4 and 7.7 mm (corresponding to the distance range of the metallic structures in each radially arranged subarray), there is always one vortex phase singularity with the same handedness presented in the 2 to 2.2 GHz range. This ensures that when incident pulses are in the 2 to 2.2 GHz range, the radial metasurface can convert radially polarized electromagnetic waves into spatiotemporal vortices at each radial position, thereby forming a scalar toroidal vortex. Owing to asymmetric modulation and Fourier transform properties, a phase singularity in the transmission spectrum function of the radial metasurface unit at point $\left(k_{r0},\omega_{0}\right)$ in the $\left(k_{r},\omega \right)$ domain can be directly translated into the space-time (r,τ) domain for the transmitted pulse.

### Scalar and vector toroidal vortex performances of generated HETVs

**Fig. 3.**
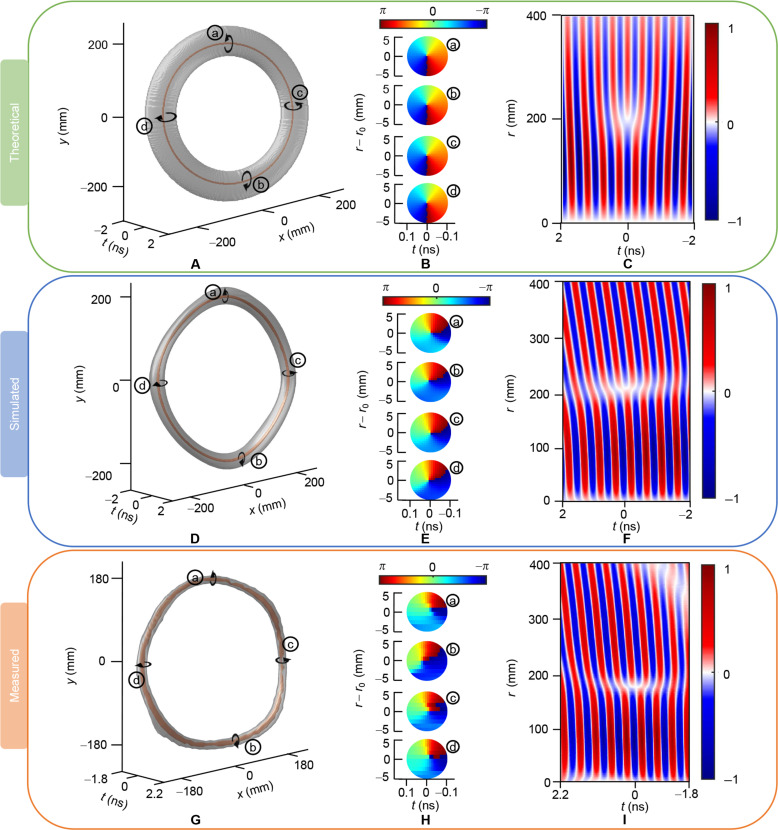
Intensity and phase characterization of HETVs. (**A**) Theoretical, (**D**) simulated, and (**G**) measured 3D iso-intensity profile of the radial polarized electric field components of the toroidal vortex pulse. The outer layer of the isosurface is depicted in gray, while the vortex core surface is highlighted in brown for clarity. The outer gray isosurfaces have a normalized level of 0.3, 0.15, and 0.13 in theoretical, simulation, and experiment conditions respectively, while the brown vortex cores have a normalized isointensity level of 0.02, 0.03, and 0.06, respectively. Theoretical, simulated, and measured phase profiles of the four slices in local coordinates, where $r=\sqrt{x^{2}+y^{2}}$ and *r*_0_ is the radius of scalar toroidal vortex core under cylindrical coordinate, are respectively shown in (**B**), (**E**), and (**H**), revealing a 2π spiral phase. (**C**) Theoretical, (**F**) simulated, and (**I**) measured 2D spatiotemporal electric field distribution at an (r,t) plane, demonstrating the presence of a fork-shaped pattern indicating the vortex singularity.

**Fig. 4.**
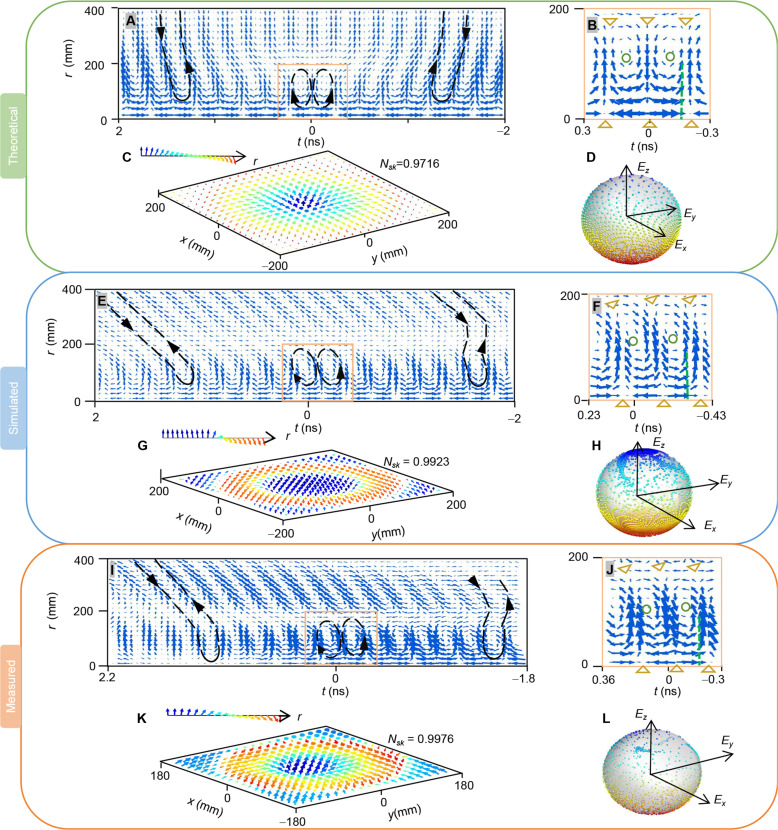
Vector field distribution of HETVs. (**A**) Theoretical, (**E**) simulated, and (**I**) measured 2D spatiotemporal electric field vector distribution at an (r,t) plane. The black dashed lines are included as visual guides. The two counter-rotating vector vortices in (A), (E), and (I) are highlighted by black dashed circles, which are further magnified in the (**B**) theoretical, (**F**) simulated, and (**J**) measured results near the vortex singularity. The unclosed black dashed lines on both sides in (A), (E), and (I) indicate that the vector electric field remains continuous along the radial direction, absent scalar toroidal singularities. Similar vector field distributions can be observable at various radii, characteristic of a vector toroidal vortex. The field displays vector singularities, such as saddle points (“longitudinal-toward radial-outward” or “radial-toward longitudinal-outward”, indicated by yellow triangles) and vortex rings extending away from the central axis (where electric vectors encircle to form a vortex loop, marked by green circles). The green dashed lines in (B), (F), and (J) denote the positions of the theoretical, simulated, and measured skyrmion textures in (**C**), (**G**), and (**K**) at specific times on the *xy* plane, respectively. The *N*_sk_ is approximately 1, indicating well-defined skyrmion textures. The coverage of the sphere of field vectors in (**D**), (**H**), and (**L**), respectively, corresponding to the skyrmion textures in (C), (G), and (K), spans the surface of the sphere, confirming the presence of skyrmions.

We demonstrate the topologically protected generation of HETVs in simulations and experiments, showcasing scalar and vector toroidal vortices schematically in Figs. 3 and 4. The coaxial horn emitter and 144 radial metasurface subarrays are mounted on a foam support structure to create the HETV generator, with subarrays along each radial direction starting from *r* = 40 mm; details are provided in Methods and the Supplementary Materials (note S3). The coaxial horn emitter is driven by a Gaussian signal at carrier frequency ω_0_ = 2.1 GHz in simulations, which slightly shifts to ω_0_ = 2.3 GHz in experiments due to material parameter and fabrication errors. The bandwidth of feeding signal is 0.2 GHz. HETV generation was experimentally observed in an anechoic chamber using a planar near-field measurement system, detailed in the Supplementary Materials (notes S4 and S5).

Figure 3 (A, D, and G) presents the theoretical, simulated, and measured 3D iso-intensity profile of the radial polarized electric field components of the HETV pulse, respectively. Semitransparency highlights the ring structure and reveals the hidden ring-shaped vortex core, with the outer layer in gray and the vortex core surface in brown for clarity. The outer gray isosurfaces have a normalized level of 0.3, 0.15, and 0.13 in theoretical, simulation, and experiment conditions, respectively, while the brown vortex cores have a normalized isointensity level of 0.02, 0.03, and 0.06, respectively. To depict the spiral phase rotating around the vortex core, four slices are taken radially and their phase profiles in local coordinates, where $r=\sqrt{x^{2}+y^{2}}$ and *r*_0_ is the radius of scalar toroidal vortex core, are shown in Fig. 3 (B, E, and H). All slices exhibit a 2π spiral phase, indicating the existence of OAM. Within a local region around the OAM center, OAM per photon at the chosen radial slices corresponding to the theoretical results is consistently 1.013ℏ. In simulations, the OAM per photon at the chosen radial slices is 1.26ℏ, 1.18ℏ, 1.11ℏ, and 1.23ℏ, respectively, while in measurements, it is 1.28ℏ, 1.20ℏ, 1.22ℏ, and 1.20ℏ, respectively. See the Supplementary Materials (note S7) for the OAM calculation method. The intensity and phase profiles confirm the pulse’s nature as a scalar toroidal vortex. It is important to note that because this scalar toroidal vortex originates from a radially polarized vector wave, the spatiotemporal phase refers to radial directions at each slice. In contrast, for a linearly polarized scalar toroidal vortex, the spatiotemporal phase refers to a linear direction (*26*). Figure 3 (C, F, and I) displays the theoretical, simulated, and measured 2D spatiotemporal electric field distribution at an (r,t) plane, respectively. The overall electric and magnetic field distributions on the (*r*, *t*) and (*z*, *t*) plane and the radial spectrum distribution can be seen in the Supplementary Materials (note S6). Because of symmetry, only the positive *x*-axis region is shown, with similar field distributions on other radii detailed in the Supplementary Materials (note S5). A fork-shaped pattern around (*t* = 0, *r* = 200 mm) indicates a 2π vortex in the space-time domain, corresponding to the double-layer toroidal isosurfaces in Fig. 3 (A, D, and G) and the observed phase vortex in Fig. 3 (B, E, and H). Because of material and assembly errors, the measured results show slight differences in the positions of singularities at different slices compared to the simulated results. However, the overall scalar toroidal vortex topology is well preserved, thereby validating the effectiveness of this method.

Because the toroidal parameter in scalar electromagnetic toroidal vortices is phase based, the pulses with these vortices are narrowband, contrasting sharply with the ultrawideband pulses required to observe vector toroidal pulses (*20*, *32*, *33*). When narrowband signals are fed into an isolated coaxial horn emitter without radial metasurfaces, the radiated wave’s electric field fails to form a proper vector toroidal vortex near the propagation axis, as detailed in the Supplementary Materials (note S9). However, in the proposed design of a coaxial horn emitter with radial metasurface, the singularities of the scalar toroidal vortex and radial polarization can induce the formation of vector toroidal vortices, as illustrated in Fig. 4.

From Fig. 3 (C, F, and I), it can be observed that at the positions of the fork-shaped pattern, the radial polarized electric field components of the pulses exhibit opposite signs around the vortex singularity, leading to the formation of saddle points at those locations and consequently generating vector toroidal vortices together with saddle points accompanied by radial polarization along the propagation axis, as depicted in Fig. 4. Figure 4 (A, E, and I, respectively) illustrates the distribution of theoretical, simulated, and measured electric field vectors on the same (*r*, *t*) cross section shown in Fig. 3 (C, F, and I). At distant times from the saddle point induced by scalar vortex singularity (*t* = 0), the vector electric field maintains continuously off the propagation axis, with no saddle points outside the propagation axis delineated by the unclosed black dashed loop in the two sides in Fig. 4 (A, E, and I). At and near the scalar vortex singularity, within several half-wavelengths, saddle points at scalar vortex singularity and propagation axis form two counter-rotational vector vortices emphasized by two black dashed circles with opposite arrows in Fig. 4 (A, E, and I) around *t* = 0, which is further highlighted in the enlarged view: Fig. 4 (B, F, and J) with the saddle points represented by yellow triangles and the vector vortex center represented by green circles Because of the nearly rotational symmetric structure of the coaxial horn with radial metasurface used, similar vector field distributions are observed at different radii, detailed in the Supplementary Materials (note S5). Consequently, the spatiotemporal vortex singularities generated by radial metasurfaces induce HETVs, coupling scalar and vector toroidal vortices in topological structure and spatiotemporal positioning. As a contrast, when the coaxial horn without radial metasurfaces is fed by the same pulses with the same bandwidth, neither scalar nor vector toroidal vortices can be observed near the propagation axis; see the Supplementary Materials for details (note S9).

Similar to vector toroidal vortices, HETVs also feature skyrmion topology, illustrated in Fig. 4 (C, G, and K). These skyrmion textures vary across different transverse planes near the center of the electromagnetic vortex ring while maintaining a Néel-type helicity. The skyrmion number (*N*_sk_) alternates between “±” on either side of the saddle points. Both experimental and simulated HETVs consistently exhibit a *N*_sk_ of approximately ±1, confirming the presence of skyrmion textures. The comprehensive coverage of the field vector sphere (*38*) in theoretical, simulated, and experimental HETVs provides further validation of the skyrmion presence, as shown in Fig. 4 (D, H, and L, respectively), with detailed calculation methods outlined in the Supplementary Materials (note S8). The vector field results demonstrate that both the simulated and measured data align well with the theoretical predictions, clearly exhibiting an HETV topology with skyrmion textures.

**Fig. 5.**
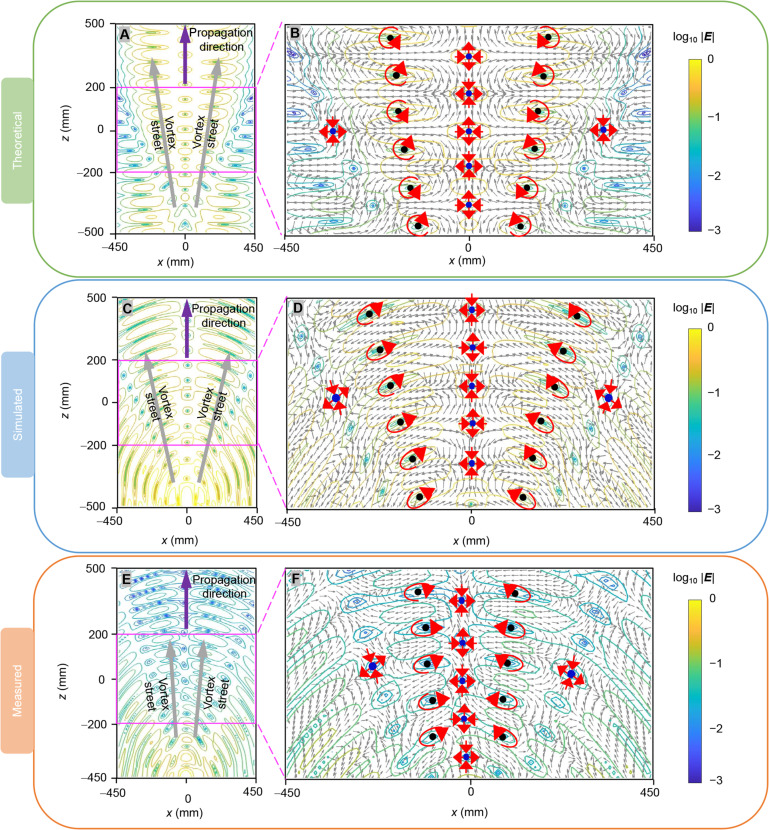
Vortex streets within HETVs. Around the scalar vortex singularity, within several half-wavelengths, the interconnected vector vortices indicate the formation of a vortex street along the propagation direction. This is visualized using contour lines on the *zx* plane at *t* = 0 in (**A**) theoretical, (**C**) simulated, and (**E**) experimental results. Enlarged views of the vortex street vector field distributions are shown in (**B**), (**D**), and (**F**), where the interconnected counter-rotational vector vortices are highlighted with red circular arrows. Their cores are marked by black dots, while the saddle points are represented by blue dots. The blue dots marked in vortex streets off the propagation axis are exactly the saddle points induced by a scalar toroidal vortex. Because of experimental constraints, measurement in the *z* direction is limited to a range of 950 mm.

Within several half-wavelengths around the vortex singularity, multiple vector toroidal vortices are interconnected along the propagation direction, forming an electromagnetic vortex street, a phenomenon previously theoretically predicted in propagating space-time electromagnetic supertoroidal pulses (*23*). However, electromagnetic vortex streets have yet to be experimental observed, as supertoroidal pulses exhibit much more complex spatiotemporal waveforms and spectra compared to the toroidal pulses already generated at optical (*20*), terahertz (*32*), and microwave (*33*) frequencies. An insight view of vortex streets with vector distributions in theory, simulation, and experiment is given in Fig. 5, where the interconnected counter-rotational vector vortices are emphasized by red circular arrows with their vortex cores denoted as black dots. Notably, blue dots marked in vortex streets off the propagation axis are exactly the saddle points induced by a scalar toroidal vortex. Vortex streets in fluids typically appear behind obstacles, with the vortices gradually weakening as the distance between the vortices and the obstacle increases. Similarly, electromagnetic vortex streets in HETVs emerge around the scalar vortex singularities, and as *z* increases—i.e., as the distance between the scalar vortex singularity and the induced saddle points increases—the saddle point phenomenon on both sides gradually weakens. This phenomenon is consistent with that observed in Fig. 4 (A, E, and I), where the vector electric field remains continuous along the radial direction as it moves away from the scalar toroidal vortex singularities. The observation demonstrates that an electromagnetic vortex street can form within HETVs, thus circumventing the challenges associated with generating supertoroidal pulses to create such topological textures.

## DISCUSSION

In conclusion, we propose a class of electromagnetic pulse solutions, known as HETVs. These topological pulses exhibit peculiar electromagnetic features, such as vortex streets, skyrmions, and transverse OAM. We demonstrate a method for their generation using coaxial horn antennas equipped with radial metasurface. Our investigation into the performance of scalar and vector toroidal vortices in this proposed scheme confirms the successful generation of HETVs.

Building on this approach, there is potential to extend the concept to other types of HETVs. Specifically, the scheme can potentially generate transverse-electric microwave toroidal pulses using an azimuthally polarized coaxial horn antenna with circumferential metasurfaces. The realization of an azimuthally polarized coaxial horn could involve substituting the inner and outer conductors with artificial magnetic conductors. Furthermore, this approach can be expanded into the terahertz and optical frequency ranges. Toroidal pulses have already been generated in these frequency ranges (*20*, *32*), and recent advancements in asymmetric metasurfaces or metagratings offer pathways to generate optical and acoustic spatiotemporal vortices (*28*–*31*). Integrating these vector toroidal pulse generation devices with spatiotemporal scalar vortex generation surfaces can facilitate the production of HETVs in corresponding frequency bands. Notably, the proposed excitation method demonstrates notable robustness to loss, suggesting that such HETVs are likely to persist even under conditions of increased optical losses. For a detailed discussion on this aspect, refer to the Supplementary Materials (note S10).

The unique characteristics of HETVs open up opportunities in the field of electromagnetics, particularly for applications in structured wavefront engineering and topologically nontrivial light-matter interactions. The topology-protected nature of spatiotemporal vortices (*32*) and skyrmions (*39*) makes them resistant to certain types of disturbances. Consequently, HETVs, which incorporate these topology-protected features, may present advantages when propagating through perturbations. In addition, fundamental-mode vector toroidal vortices can excite toroidal modes and anapoles in matter (*24*). HETVs with electromagnetic vortex street features have the potential to stimulate high-order toroidal multipoles in matter or generate intricate interactions with toroidal metamaterials (*40*–*42*). Moreover, the phase and vector vortices (*43*) in the longitudinal plane, along with the skyrmion texture with subwavelength features (*44*) in the transverse plane, make HETVs highly promising for achieving high-precision sensing and imaging.

## METHODS

### Fabrication and measurement method

Each metasurface subarray with 2 units was fabricated using the printed circuit board technique. The substrate used has a thickness of 1.5 mm, accompanied by a relative dielectric constant of 16 and a loss tangent of 0.001. The coaxial horn emitter’s configuration and dimensions are congruent with those outlined in (*33*). To assemble the HETV generator, the coaxial horn emitter is mounted alongside 144 radial metasurface subarrays onto a foam holder. These subarrays are positioned along each radial direction, commencing from a radius of 40 mm. We used a planar microwave anechoic chamber for comprehensive measurements of the spatial electromagnetic fields emitting from the HETV generator. By interconnecting a vector network analyzer to both the HETV generator and the probes, we procured transmission response measurements, enabling us to discern the magnitude and phase signatures of the electromagnetic field across diverse spatial locales. To accurately capture both transverse and longitudinal electric field components, we used a rectangular waveguide probe and a monopole probe, respectively. For a detailed account of our methodology, refer to the Supplementary Materials (notes S2 to S4). During the transverse component measurements, we precisely adjusted the polarization direction of the rectangular waveguide probe to align with the radial axis of the HETV generator. Similarly, for the longitudinal component assessments, we aligned the polarization of the monopole probe with the axial direction of the generator. Our scanning system was programmatically orchestrated to traverse the designated plane, facilitating the acquisition of a comprehensive field distribution. By harnessing the frequency domain measurement method outlined, we extracted the spatial magnitude and phase characteristics of the HETV generator. Subsequently, we used inverse Fourier transformation to reconstruct the time-domain field; see the Supplementary Materials (note S5) for details. The synthesis of the transverse and longitudinal electric field components within the spatiotemporal field distribution enabled us to construct a comprehensive scalar and vector field representations, offering profound insights into the electromagnetic behavior of the HETV generator.
