# Supplementary material for: Hybrid electromagnetic toroidal vortices
Source: Sci Adv. 2025 Feb 21;11(8):eads4797. doi: 10.1126/sciadv.ads4797 (PMC11844730; doi:10.1126/sciadv.ads4797)
Supplement: Supplementary file 1 — Supplementary Text Figs. S1 to S11 [file sciadv.ads4797_sm.pdf]

Science Advances
AAAS

Supplementary Materials for

Hybrid electromagnetic toroidal vortices

Ren Wang *et al.*

Corresponding author: Ren Wang, rwang@uestc.edu.cn; Musheng Liang, msliang@uestc.edu.cn; Yijie Shen, yijie.shen@ntu.edu.sg

Sci. Adv. **11**, eads4797 (2025)
DOI: 10.1126/sciadv.ads4797

This PDF file includes:

Supplementary Text
Figs. S1 to S11

Supplementary Text

Note S1: Theoretical derivation of hybrid electromagnetic toroidal vortices (HETVs).

Fourier transformations (FTs) between the time and frequency domains, as well as between the space and spatial frequency domains, provide substantial advantages in transforming unbounded or large-scale time and space regions into simplified frequency and spatial frequency regions. The conservation of orbital angular momentum (OAM) within this 2D-FT process, from the time domain to the frequency domain, has been demonstrated by (*27*). For an arbitrary radial calculation range, the transfer function of the electromagnetic wave conversion structure can be expressed as (*37*):

$$H(k_r,\omega) \approx S_r k_r / k_0 + S_t(\omega - \omega_0)/\omega_0 , \tag{S1}$$

where k_r is the transverse wave vector along the radial direction, ω is the angular frequency of a plane wave centered at ω_0 with a wave vector k_0, and S_r and S_t are two complex constants determining the amplitude and phase distribution in the $k_r - \omega$ domain. Ideally, the amplitude of the transfer function remains constant, and a 2π spiral phase is uniformly distributed along the azimuthal direction in the $k_r - \omega$ plane. In this case, the amplitudes of S_r and S_t can be normalized to 1, and a π/2 (OAM mode: l=−1) or −π/2 (OAM mode: l=+1) phase difference is required between S_r and S_t. Through 2D-FT, the transfer function in the spatiotemporal domain can be expressed as:

$$h(r,t) \approx \iint H(k_r,\omega)\exp(i(\omega-\omega_0)t + ik_r r)dk_r d\omega . \tag{S2}$$

The spatial distribution of the radial component of the incident wave can be represented by

$$s(r) = \frac{r}{z_0\sqrt{2\pi}}\exp(-\frac{r^2}{z_0{}^2}) \,, \tag{S3}$$

where $s(r)$ follows a Gaussian distribution along the radial direction, and z_0 is a constant related to the field intensity distribution. The space-domain convolution of $s(r)$ and $h(r, t)$ leads to the output radial polarization field. At a specific radial position, there is only one pulse for both $s(r)$ and $h(r, t)$, meaning that the radial component envelope $\Psi(r,t)$ of the output wave can be directly given by the product of the radial polarization factor of the incident wave and the transfer function as:

$$\Psi(r,t) = s(r)h(r,t) \,. \tag{S4}$$

The fully radially polarized output electromagnetic field $E_r(r,t)$ can be expressed as:

$$E_r(r,t) = \Psi(r,t)\exp(-i\omega_0 t + ik_0 z) \,, \tag{S5}$$

where z is the propagation distance, which should be chosen specifically to observe the generated HETVs. The longitudinal component of the generated wave in cylindrical coordinates can be determined according to Gauss's law (*20*), as follows:

$$E_z(r,z) = -\int_{\alpha}^{z}\frac{E_r(r,z^{'})}{r} + \frac{\partial E_r(r,z^{'})}{\partial r}dz^{'} \,, \tag{S6}$$

where α is a reference point with zero field.

In our experiment, $\omega_0 = 2\pi \times 2.1 \times 10^9$ rad/s, $(\omega - \omega_0)/\omega_0 \in [-0.1, 0.1]$, $k_r / k_0 \in [-0.1, 0.1]$. Applying Eqs. (S1–S5), the scalar toroidal vortex field can be delineated. The theoretical 3D iso-intensity profile in Fig. 3 describes the radial polarization component comprising the scalar toroidal vortices near the vortex cores. Further insight into these scalar toroidal vortices reveals the phase patterns

of four radial cross-section slices, numbered a–d, surrounding their local vortex cores, where the 2π phase spirals demonstrate OAM vortices with topological charge of 1. Additionally, a 2D fork-shaped field pattern in the $r-\tau$ plane is shown in Fig. 3. The opposite field signs neighboring the fork's cross imply a 2π vortex generated in the spatiotemporal domain, forming a saddle point at the vortex singularity. Furthermore, vector fields can be composed based on the calculated E_r and E_z components from Eq. (S6). The saddle points generated by vortex singularities in scalar toroidal vortices ensure that theoretical vector toroidal vortices can be obtained, as shown by the 2D vector field in Fig. 4, with the same cross-section in the $r-\tau$ plane as the scalar field. Vector vortices disappear at the observing plane, with the field reconstructing after the fork-shaped field period. The coexistence of scalar and vector toroidal vortices results in the desired HETVs.

Note S2: Metasurface unit design.

The design of the metasurface unit begins by examining its phase and amplitude responses within the transmission spectrum function $T(k_x,\omega)$, where k_x is the wave vector component along the unit's x-axis, determined by the incident wave angle, and ω is the angular frequency of the plane wave. To exhibit a phase spiral in the $k_x-\omega$ plane, the phase responses of the unit under different incident angles is asymmetric, necessitating an asymmetrical design of the metasurface unit. Under the unit cell boundary conditions along the *x* and *y* directions, as depicted in Fig. S1**A**, and for a range of incident angles near normal incidence (−10° to 10°), the metasurface unit can be simulated in CST software to obtain the transmission spectrum function.

The asymmetric metasurface unit we use is depicted in Figs. S1**A** and **B**. Its asymmetry is characterized by two oppositely oriented C-shaped copper foils that are shifted along the unit, as

described by parameter *e* in Fig. S1**A**. Identical copper foils are mounted on the back of the substrate to ensure symmetry in the overall radial metasurfaces. The designed parameters are a = 12.8, b = 19.3, c = 7, d = 14.2, e = 19.3, w = 14, h = 1.5, l = 85 (unit: mm). The substrate of the metasurface unit is TP-2, with a dielectric constant of 16 and a loss tangent of less than 0.001.

To illustrate the impact of asymmetry, metasurface units with copper foils shifted by e=0 mm, 9.8 mm, and 14.2 mm were simulated with distance between neighbored units as p=4.5 mm. The corresponding metallic slabs are shown in Fig. S1**C**–**E**, and their transmission responses are given in Fig. S1**F**–**K**. As the foil shift increases, the phase distribution of the transmission spectrum function becomes increasingly asymmetric and gradually exhibits a 2π spiral distribution.

Note S3: Structure and fabrication of HETV generator.

To facilitate actual measurement, a support structure is necessary to secure the 144 discrete radially distributed metasurface subarrays and ensure that both the coaxial horn emitter and the radial metasurface are aligned along the same central axis, as illustrated in Fig. S2**A**. The front side of the support, shown in Fig. S2**B**, is designed to accommodate the 144 radial metasurface subarrays. This support structure features a hollow ring with an inner radius r = 40 mm and a width l = 85 mm, which correspond to the starting radius of the radially displayed metasurface subarrays and the length of a single metasurface unit along the radial direction. Additionally, the structure includes 144 radially oriented slots, each with a width h = 1.5 mm and length l = 85 mm, matching the thickness and radial length of the metasurface unit substrate, to hold the 144 radial metasurface subarrays. Due to the limited manufacturing precision of the support structure, only the outer metasurface units are inserted into the slots.

The back side of the support, depicted in Fig. S2**C**, is designed to hold the coaxial horn emitter. The central circle shown in Fig. S2**C** represents the right view of a cylindrical hollow on the back side, with a diameter d = 260 mm. A comprehensive view of all the fixing parts of the support is provided in Fig. S2**E**. The height of the cylindrical hollow on the back side is hz = 59 mm, ensuring that the distance between the coaxial horn emitter and the radial metasurface matches that in the simulation. The height of the slots on the front side, as shown in Fig. S2**F**, is w = 14 mm, which is equal to the width of the metasurface units along the propagation direction and ensures that the metasurface subarrays are aligned on the same plane. The overall size of the support is $ws \times ws \times hs = 520 \times 520 \times 100$ mm^3. The support structure is fabricated using 3D printing with foam (ε_r = 1.45), and the radial metasurface subarrays are produced using PCB technology.

Note S4: Experimental setup and measurement method.

The setup for measuring the emitting electric field of the HETV generator is shown in Fig. S3. We measured the spatial electromagnetic field of the HETV generator in a planar microwave anechoic chamber, where the probe is mounted on a scanning frame. This allows the probe to move with the frame, enabling measurements of the electric field at any desired location in space. The scanning system is programmatically controlled to scan within the required plane, allowing us to capture the field distribution in the target plane. The HETV generator is fixed on a transmission stand during these measurements.

We used an R&S®ZNA vector network analyzer to measure the S_{21} parameters between the HETV generator and probe, capturing the amplitude and phase characteristics at different spatial positions. This vector network analyzer supports a wide frequency range of 10 MHz to 50 GHz.

Our target measurement band is 2.1–2.3 GHz, with measurement points spaced 50 MHz apart. For the transverse polarization components of the electric field (i.e., the *x* and *y* components), we employed a waveguide probe that supports a frequency range of 1.7–2.8 GHz. For the longitudinal polarization component (i.e., the *z* component), we used a monopole antenna, which supports a frequency range of 1.4–10.5 GHz. Both probes cover our target measurement band. To better illustrate the overall radiation characteristics of the antenna array, we measured the distribution of the longitudinal component and the two transverse components within a 1-square-meter target area, with a sampling interval set at 0.01 m.

Note S5: Spatiotemporal field calculation method from the measured spectral distribution.

The measured result is the system's transfer function, to which a Gaussian distribution is applied. This product yields the output frequency-domain field, which can then be converted into the time domain using inverse discrete 2D-FT:

$$s(r,\theta,t)=\sum_{i=1}^{N} S(r,\theta,f_i)\exp(i2\pi f_i t) \tag{S7}$$

where *s* represents the time-domain field, *S* is the measured frequency-domain field, (r, θ) denotes a position within the detection field in polar coordinates with the origin at the field center, *t* is an arbitrary moment in time, and *N* is the number of discrete frequencies. In FT, a finite-length frequency-domain signal is transformed into a periodic time-domain signal, implying that a complete time-domain signal of the system's response can be obtained by selecting a random time period, provided the time range used in the FT operation is sufficiently long.

The amplitude and phase patterns of both the radial and longitudinal polarization fields (E_r and E_z) at 2.15, 2.2, and 2.25 GHz within the detection region are shown in Fig. S4. For the radial field, Figs. S4**A**–**C** consistently exhibit a null field around r = 180 mm, while the phase patterns near these field nulls, shown in Figs. S4**D**–**F**, display abrupt jumps of 2.57, 3.87, and 3.04 radians, respectively. These characteristics indicate the generation of scalar toroidal vortices in the spatiotemporal domain. In contrast to the radial field distribution, the longitudinal field exhibits non-zero amplitude near the radial field nulls, as shown in Figs. S4**G**–**I**, suggesting the formation of vector toroidal vortices in the spatiotemporal domain.

The time-domain fields, radially and longitudinally polarized and obtained via the FT operation, are then used to synthesize the vector toroidal vortices. To accurately depict a vector field in the spatiotemporal domain, the field observed at an earlier time should be positioned on the right side of the vector field, which can be intuitively understood by considering wave propagation.

Three additional scalar and vector spatiotemporal fields corresponding to the radial slices b, c, and d selected in Fig. 3 are presented in Fig. S5. The scalar and vector fields in these slices are consistently similar in both spatial and temporal domains, resulting in a toroidal structure of the generated scalar and vector fields.

Note S6: Temporal and spectral shape of the pulse.

Since the scalar toroidal vortex is manifested in the transversely polarized field, we present the electric field on both transverse and longitudinal spatiotemporal planes, as shown in Fig. S6. The pulse is finite in all r, z, and t domains. Scalar vortices are observed in both the r-t and z-t planes.

In the *z*-*t* plane, the waveform exhibits inclined stripes, indicating that the pulse undergoes several periods in the *z*-direction.

The magnetic field on both transverse and longitudinal spatiotemporal planes is shown in Fig. S7. Since measuring the magnetic field in the propagation space is challenging, only the theoretical and simulated results are presented. As the discussed HETVs are transverse magnetic (TM) pulses, the magnetic field consists solely of the azimuthally polarized component. The magnetic field exhibits a distribution similar to that of the transverse electric field, with scalar vortices observed in both the *r*-*t* and *z*-*t* planes.

The spectral distribution of the radially polarized electric field along the radial direction at different propagation distances is shown in Fig. S8. Due to fabrication and assembly errors, the measured spectrum shifts slightly from the simulated range of 2–2.2 GHz to approximately 2.2–2.4 GHz. In the experimental, simulated, and theoretical results, a dark region appears at the center of each spectrum, corresponding to the singularity in Fig. 2 in the main text, indicating the generation of a scalar spatiotemporal toroidal vortex.

Note S7: Calculation of orbital angular momentum.

The OAM density $\boldsymbol{j}$ is expressed as:

$$\boldsymbol{j} = \boldsymbol{r} \times \boldsymbol{p}, \tag{S8}$$

where $\boldsymbol{r}$ is the polar position vector originated from OAM center and $\boldsymbol{p}$ is the linear momentum density of corresponding scalar field u given by (*26*):

$$\boldsymbol{p} = u^* \nabla u = I \nabla \phi , \tag{S9}$$

where the asterisk (*) denotes the complex conjugate, ∇ represents the gradient operation, I and ϕ are the intensity and phase pattern of the scalar field u, respectively. Based on OAM density, OAM per photon (OAM/p) within a calculation region S can be expressed by the ratio of total OAM to the total field intensity:

$$OAM / p = \frac{\hbar \int \boldsymbol{j} dS}{\int I dS} . \tag{S10}$$

According to Eq. (S8), the direction of OAM is perpendicular to both $\boldsymbol{r}$ and $\boldsymbol{p}$, following the right-hand screw rule. For OAM of generated HETV, its OAM is oriented along ϕ direction in a cylindrical coordinate and perpendicular to the propagation direction, indicating transverse OAM. Within a local region around OAM center, OAM per photon calculated with Eq. (S10) at the chosen radial slices corresponding to the theoretical results is consistently $1.013\hbar$. In simulations, the OAM per photon at the chosen radial slices is $1.26\hbar$, $1.18\hbar$, $1.11\hbar$, and $1.23\hbar$, respectively, while in measurements, it is $1.28\hbar$, $1.20\hbar$, $1.22\hbar$, and $1.20\hbar$, respectively. As derived from Eqs. (S8-S10), OAM per photon is position-dependent, implying that an imperfect phase spiral distribution and uneven intensity distribution can cause the OAM per photon to deviate from the ideal value.

Note S8: Calculation method of the sphere of directions and skyrmion number (N_{sk}).

The topological features of a two-dimensional skyrmion model are defined by the N_{sk}, which is a key topological invariant. This number is calculated by integrating the topological charge density over the spatial domain, as given by the following equation (*38*):

$$N_{sk}(\boldsymbol{r}) = \frac{1}{4\pi}\iint \mathbf{m}(\mathbf{r}) \cdot \left[\frac{\partial \mathbf{m}(\mathbf{r})}{\partial x} \times \frac{\partial \mathbf{m}(\mathbf{r})}{\partial y}\right] \mathrm{d}x\mathrm{d}y \quad \text{(S11)}$$

where $\mathbf{m}(\boldsymbol{r}) = \frac{\boldsymbol{E}(\boldsymbol{r})}{|\boldsymbol{E}(\boldsymbol{r})|}$ denotes the unit vector field describing the skyrmion configuration, and $\boldsymbol{E}(\boldsymbol{r})$ represents the electric field at a specific location $\boldsymbol{r} = (x, y)$. The size of the integration region is chosen based on the skyrmion's spatial extent, with the boundary selected at the point where the longitudinally polarized electric field reappears for the second time. When computed using Eq. (S11), the N_{sk} typically approaches ±1, which indicates the presence of nearly integer topological charges and confirms the existence of skyrmionic structures within the electromagnetic toroidal pulses generated.

The skyrmion's vector characteristics can also be visually analyzed by mapping the vector field onto a unit sphere, a method often referred to as the "sphere of directions" (*38*). This sphere represents all possible vector directions, with each vector in the two-dimensional plane $\boldsymbol{r} = (x, y)$ mapped onto the sphere based on its orientation. By normalizing the vector length and mapping it from the origin, we can visualize whether the vectors from the entire skyrmion cell cover the sphere completely. Complete coverage of the sphere by these vectors visually indicates the presence of skyrmions, as it suggests that all possible orientations are represented within the skyrmion structure.

Note S9: Field distribution of the coaxial horn emitter fed by a narrowband signal.

Since the toroidal parameter in scalar electromagnetic toroidal vortices is phase-based, pulses with these vortices are inherently narrowband, which contrasts sharply with the ultrawideband pulses required to observe vector toroidal pulses. In fact, when narrowband signals are fed into an isolated coaxial horn emitter, the resulting electric field of the radiated wave fails to form a proper vector toroidal vortex near the propagation axis. To further illustrate the performance of the HETV generator, a spatiotemporal field simulation of the coaxial horn emitter was conducted in CST, covering the same spatial and temporal regions as those produced by the HETV generator. In this simulation, a 2–2.2 GHz pulse was fed into the emitter. The results show that at r = 0 mm along the propagation axis, the field is predominantly longitudinal and displays periodic saddle points, as depicted in Fig. S9. Unlike the field generated by the HETV generator, which shows an additional set of saddle points around r = 200 mm indicating the presence of scalar and vector vortices, the coaxial horn emitter's field does not exhibit any additional saddle points beyond r = 0 mm, suggesting the absence of such vortices.

Note S10: Performance discussion of increased loss.

The increased loss is indeed a concern at optical frequencies. To assess the sensitivity of the proposed method to loss, we varied the loss tangent of the dielectric substrate used in the metasurfaces from 0.001 to 0.05. The corresponding loss of the metasurfaces in both cases is shown in Fig. S10. As can be seen from the figure, increasing the substrate loss results in a rise in the metasurface loss from less than 0.05 to a maximum of 0.6, which is comparable to the loss typically observed in optical metasurfaces.

In the case of increased metasurface loss, the generated spatiotemporal field on a radial cross-section is shown in Fig. S11. Similar to the case with no increased loss, the distribution of radially polarized electric field component clearly exhibits Y-shaped phase singularities, and the vector field distribution shows a distinct toroidal field structure. Therefore, the proposed excitation method exhibits strong robustness to loss, indicating that such HETVs should remain present even when the system undergoes increased optical losses.

Supplementary figures

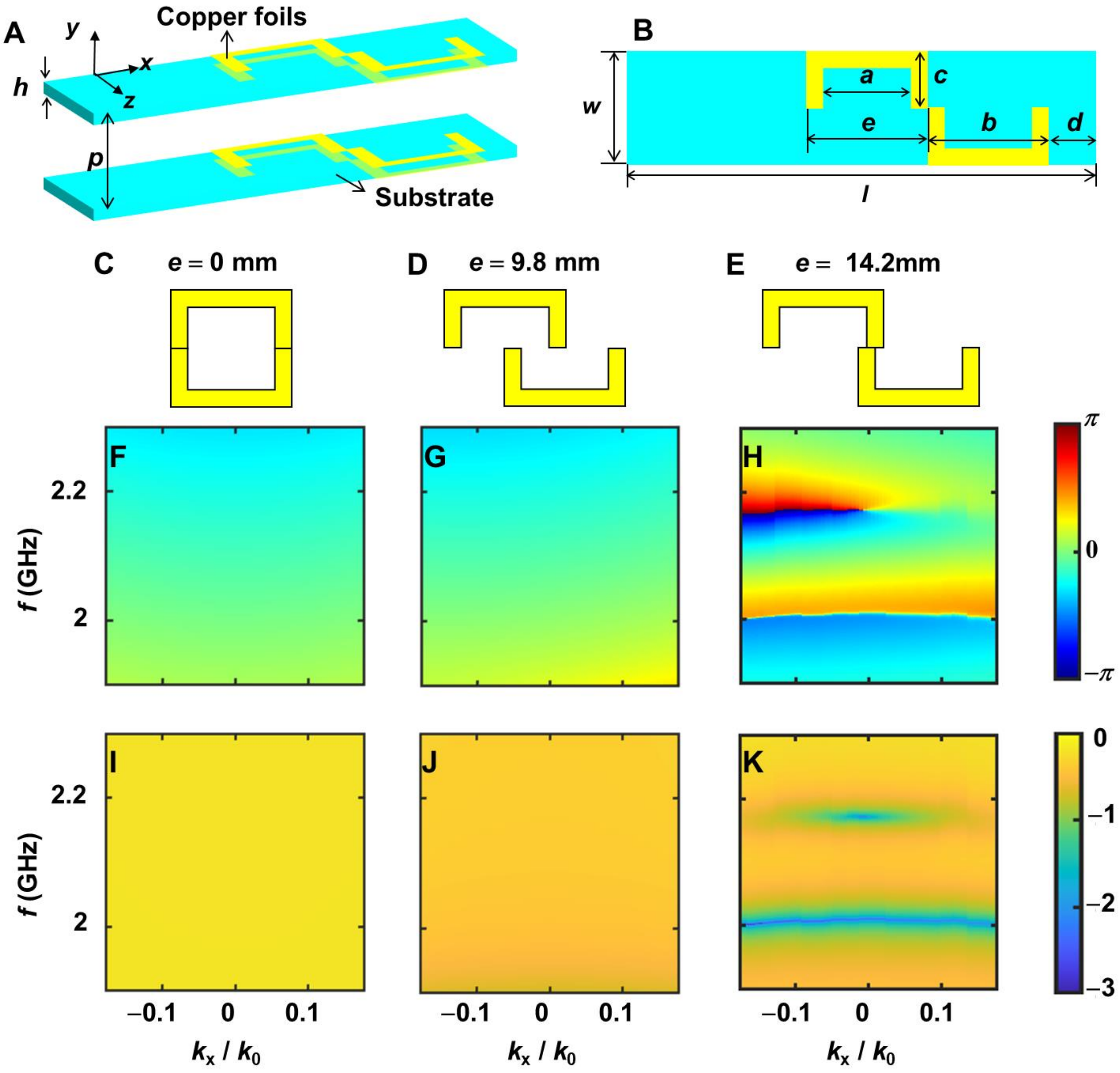

Fig. S1 Metasurface unit and its asymmetric properties illustrated by the transmission spectrum function. (**A**) 3D view of two units in an infinite unit array with period *p* and (**B**) 2D front views of designed asymmetric metasurface unit which consists a substrate layer and two mirror symmetric metallic layers, each composed of two oppositely oriented C-shape copper foils. The asymmetry of unit is featured by the shifting distance, *e* in (**B**), between two foils along the unit as the relative positions of copper foils shown in (**C**–**E**) with *e* = 0 mm, 9.8 mm, and 14.2mm

respectively. Transmission spectrum function of units with different shifting distances in (**C**–**E**): (**F**–**H**) phase responses and (**I**–**K**) amplitude responses.

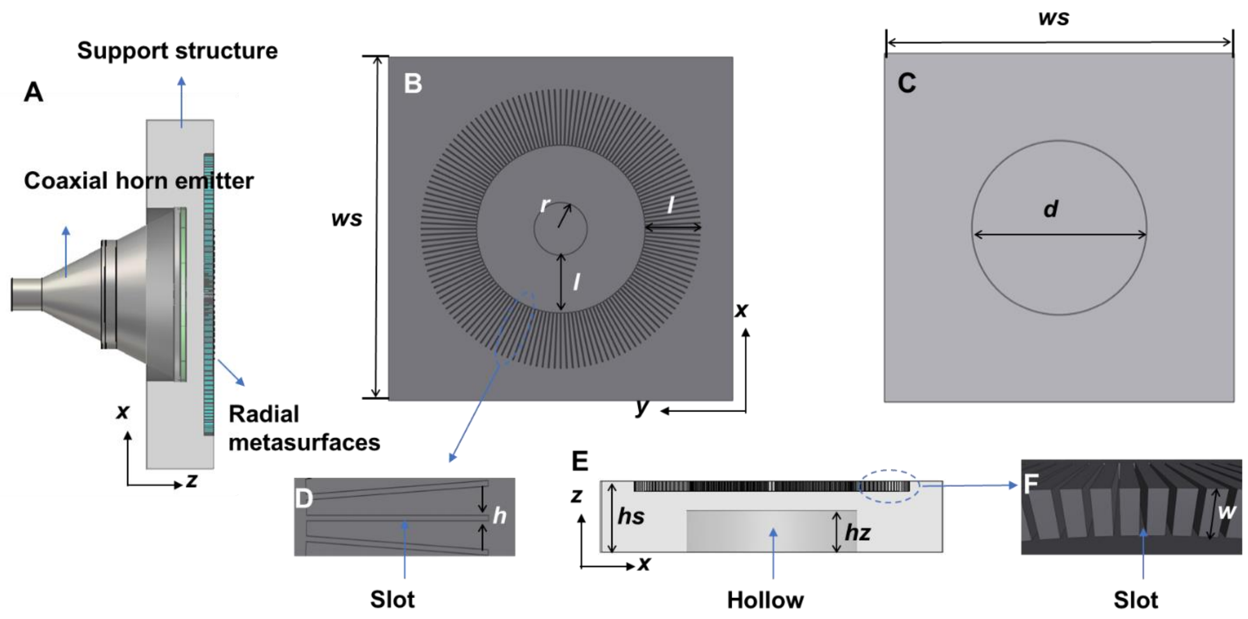

Fig. S2 Structure of the HETV generator and details of the support. (**A**) Side view of the HETV generator. Coaxial horn emitter and radial metasurfaces are inserted into the back and front side of the support structure to ensure their relative position nearly the same to that in simulation. (**B**) Front side of the support structure with a zoom in figure (**D**). The front side is designed with a hollow ring and 144 slots, which have the same geometric parameters to metasurface units in each radial direction. (**C**) The back side of the support structure has a cylindrical hollow with a diameter equals to that of the emitter. A semi-transparent side view of the support structure is given in (**E**) and an insight of slots in the front side is displayed in (**F**). The height of slots is equal to the width of metasurfaces in the propagation direction to guarantee the inserted metasurfaces almost on the same plane.

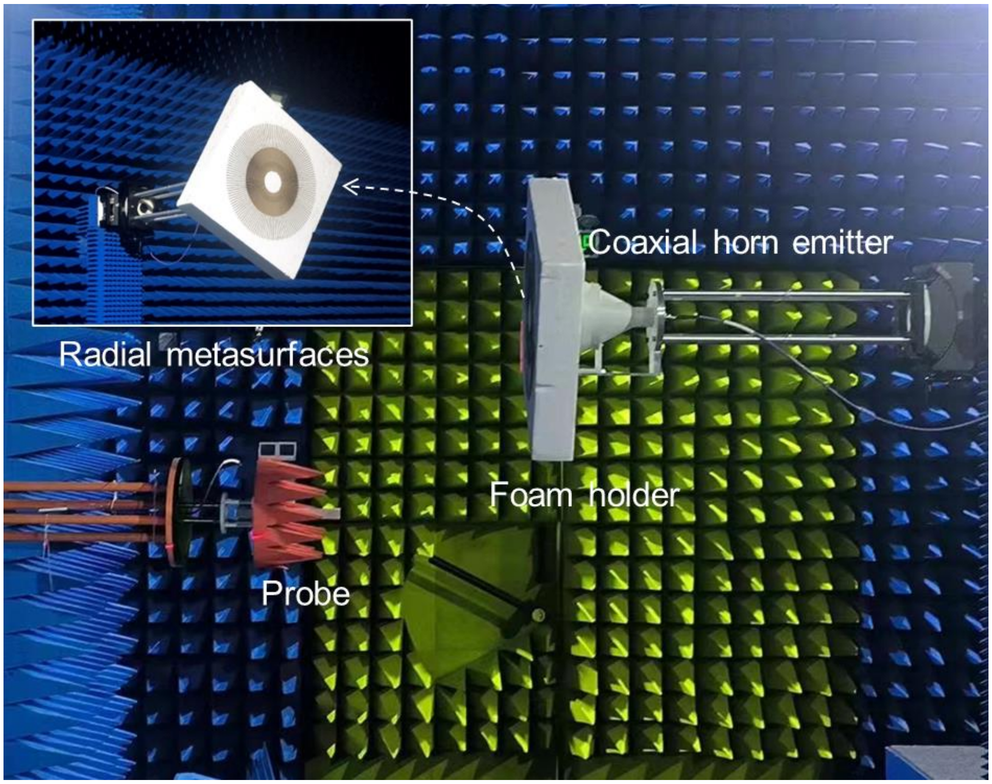

Fig. S3 Experimental setup for measuring HETV generator. The HETV generator is fixed on a transmission stand, and the probe is mounted on a scanning frame, allowing for electric field measurements at any position in space. Waveguide probes and monopole antennas are used to measure the transverse and longitudinal polarization components of the electric field, respectively.

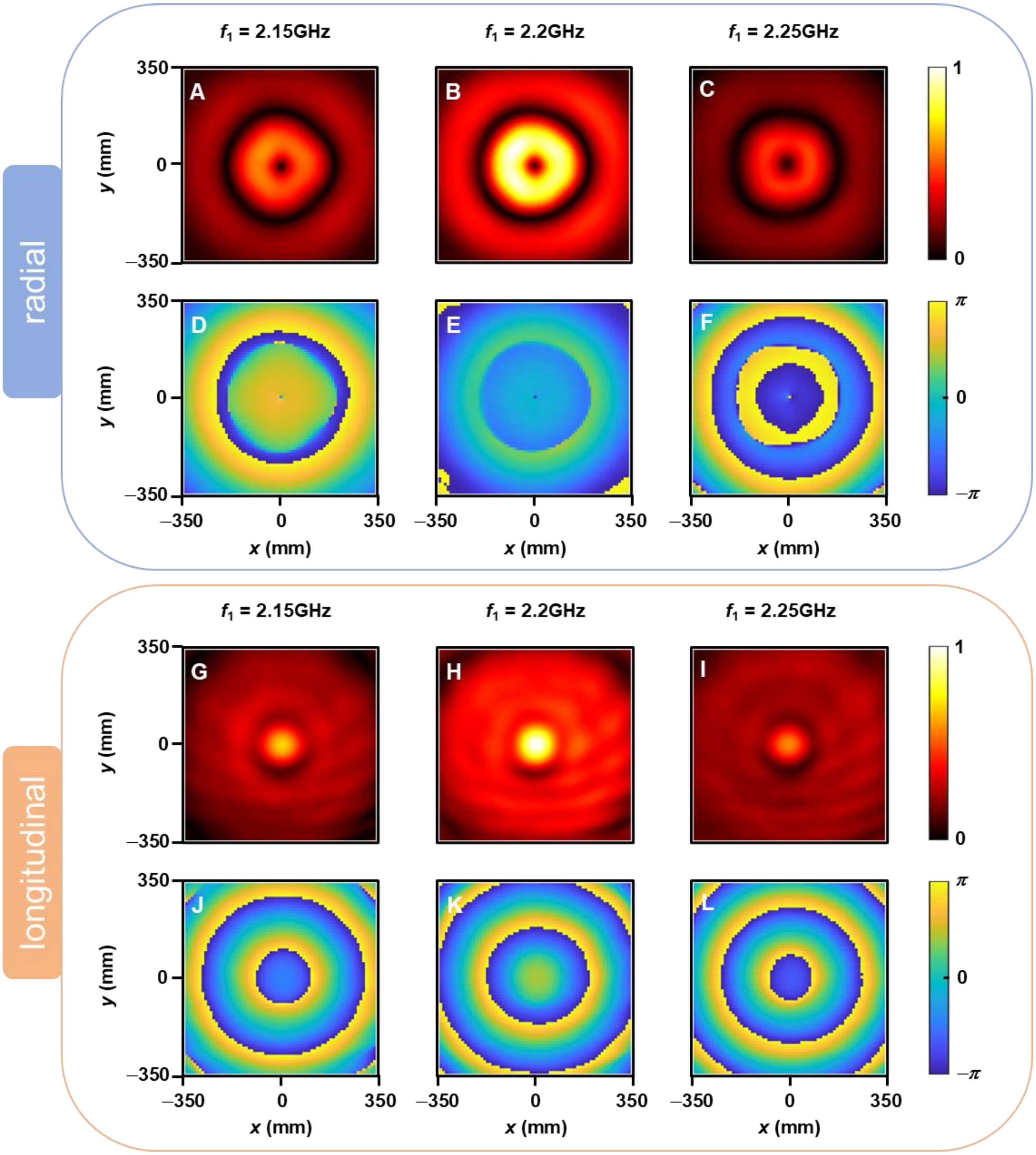

Fig. S4 Measured amplitude and phase distributions of measured frequency domain fields in both radial and longitudinal polarization. (**A**–**C**) Amplitude and (**D**–**F**) phase distributions of radial polarization fields at 2.15 GHz, 2.2 GHz and 2.25 GHz, respectively. The field nulls shown in (**A**–**C**) near r = 180 mm and abrupt phase jump neighboring field nulls at given

frequencies indicating the existence of scalar toroidal vertices in spatiotemporal domain. (**G**–**I**) Amplitude and (**J**–**L**) phase distributions of longitudinal polarization fields at 2.15 GHz, 2.2 GHz and 2.25 GHz, respectively. The longitudinal fields process local field maxima where the amplitude of radial field is approximately zero reveling the generation of vector toroidal vertices.

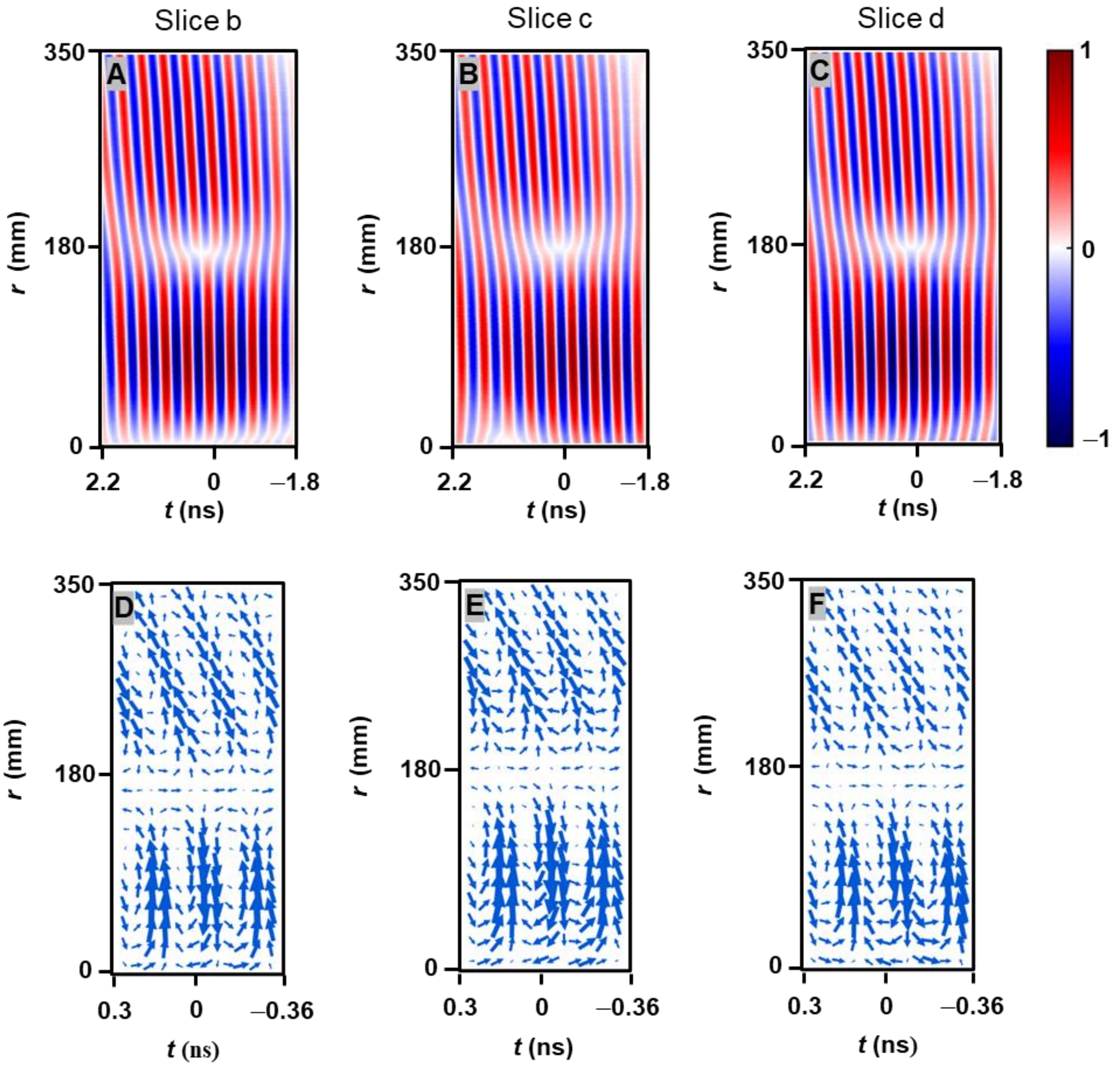

Fig. S5 Measured scalar and vector spatiotemporal fields corresponding to different radial slices in main text. (**A**–**C**) Scalar fields and (**D**–**F**) vector fields of radial slices at $-y$, $+x$ and $-x$ axes respectively. The similar distributions of both scalar and vector fields at chosen slices guarantee the toroidal shape of generated scalar and vector fields.

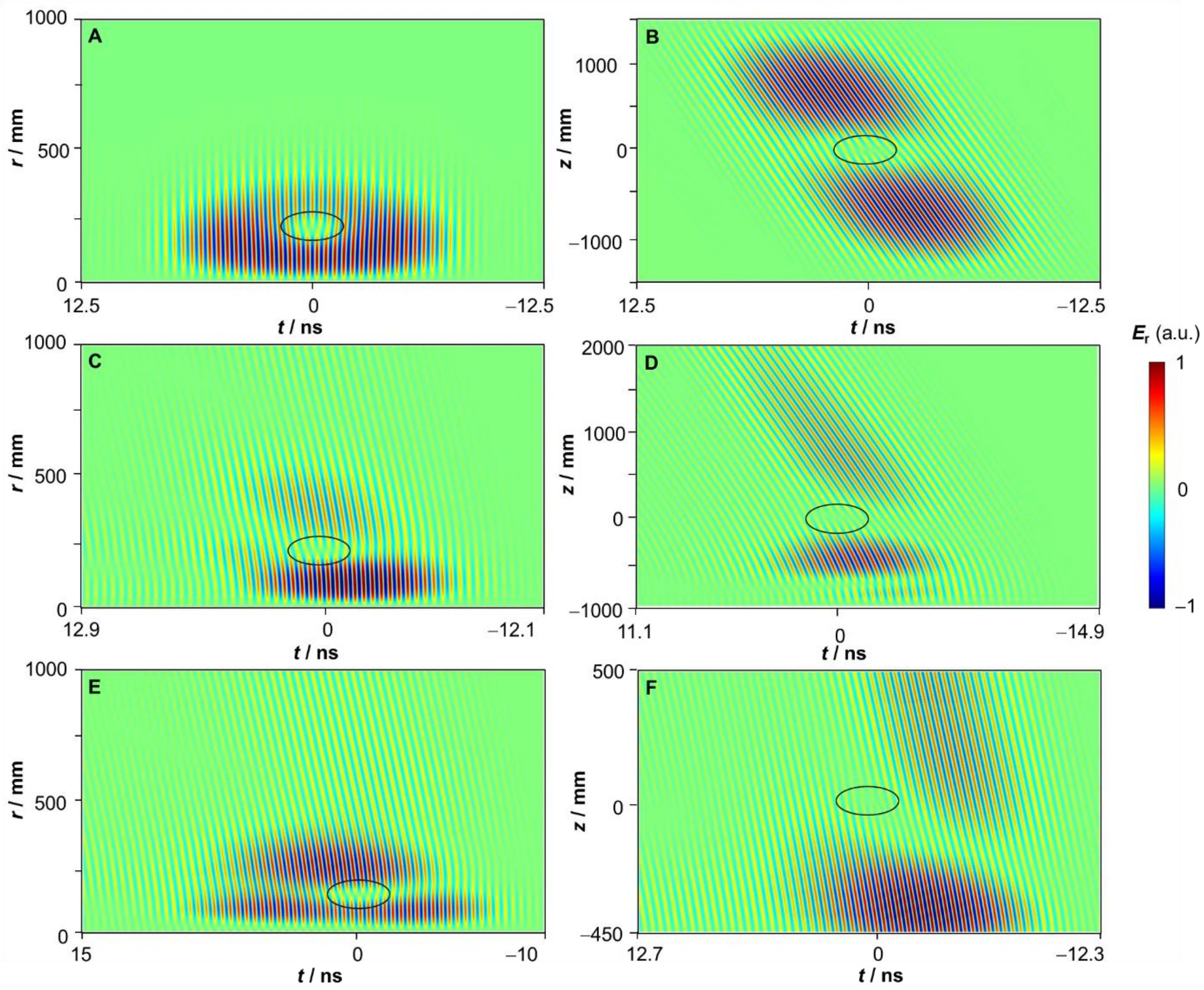

Fig. S6 Electric field on both transverse and longitudinal spatiotemporal planes. (**A**) Theoretical, (**C**) simulated, and (**E**) experimental radially polarized electric field on *r*-*t* plane. (**B**) Theoretical, (**D**) simulated, and (**F**) experimental radially polarized electric field on *z*-*t* plane corresponding to the spatial position where the scalar singularity occurred on *r*-*t* plane. Scalar vortices with Y-shaped phase singularities in both planes are illustrated in the black circles. Due to experimental constraints, measurement in the z-direction is limited to a range of 950 mm. The results within this range show good agreement with the simulation data.

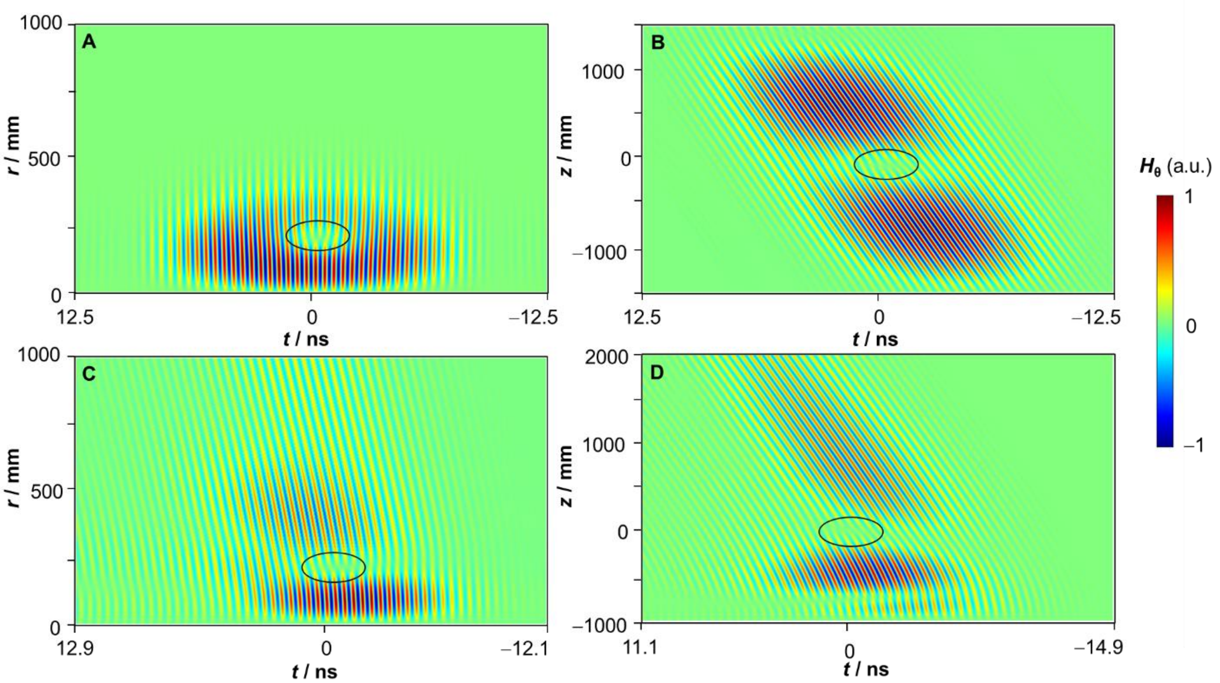

Fig. S7 Magnetic field on both transverse and longitudinal spatiotemporal planes. (**A**) Theoretical and (**C**) simulated azimuthally polarized magnetic field on *r*-*t* plane. (**B**) Theoretical and (**D**) simulated azimuthally polarized magnetic field on *z*-*t* plane corresponding to the spatial position where the scalar singularity occurred on *r*-*t* plane. Scalar vortices with Y-shaped phase singularities in both planes are illustrated in the black circles.

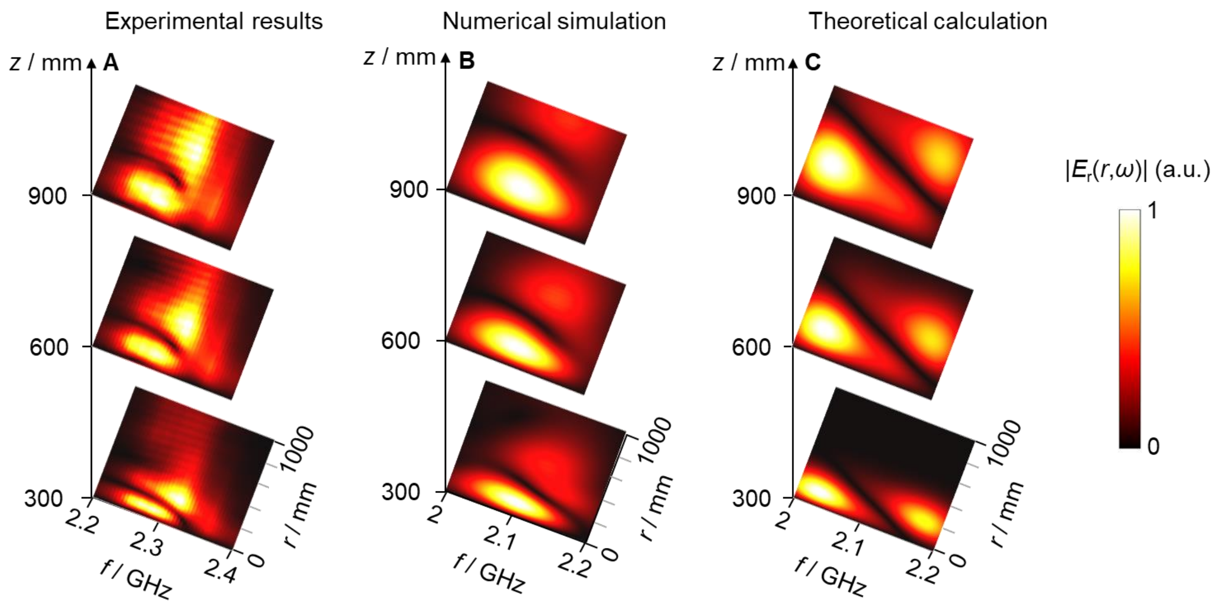

Fig. S8 Spectrum distribution of radially polarized electric field at different propagation distances. Experimental, simulated, and theoretical results are shown in (**A**), (**B**), and (**C**), respectively. A dark region appears at the center of each spectrum, corresponding to the singularity in Fig. 2 in the main text, indicating the generation of a scalar spatiotemporal toroidal vortex.

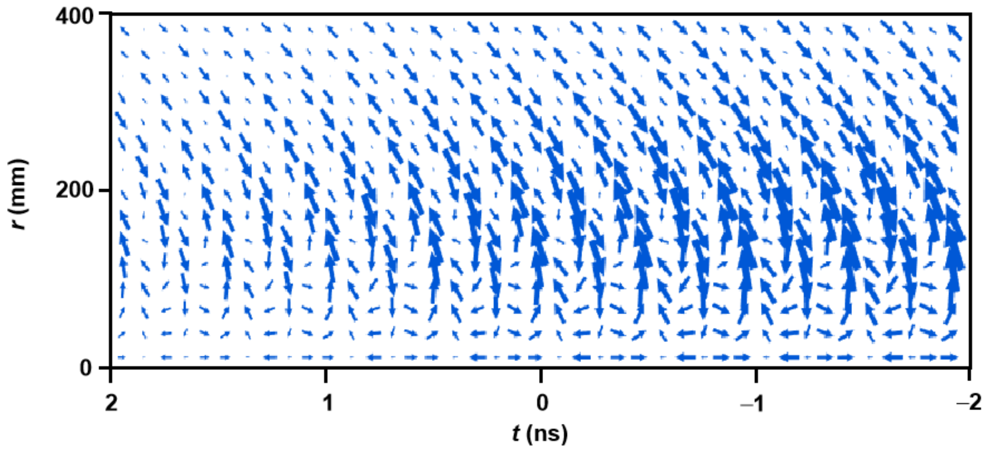

Fig. S9 Simulated vector field distribution of the coaxial horn emitter without radial metasurfaces. The coaxial horn emitter is fed by narrowband signals with the bandwidth of 2-2.2 GHz and the field is detected in the identical spatiotemporal region to that generated by HETV generator. Longitudinal field along propagation axis at r = 0 mm shows the saddle points periodically. On the other hand, there is no additional saddle points besides r = 0 mm to form the scalar and vector vertices.

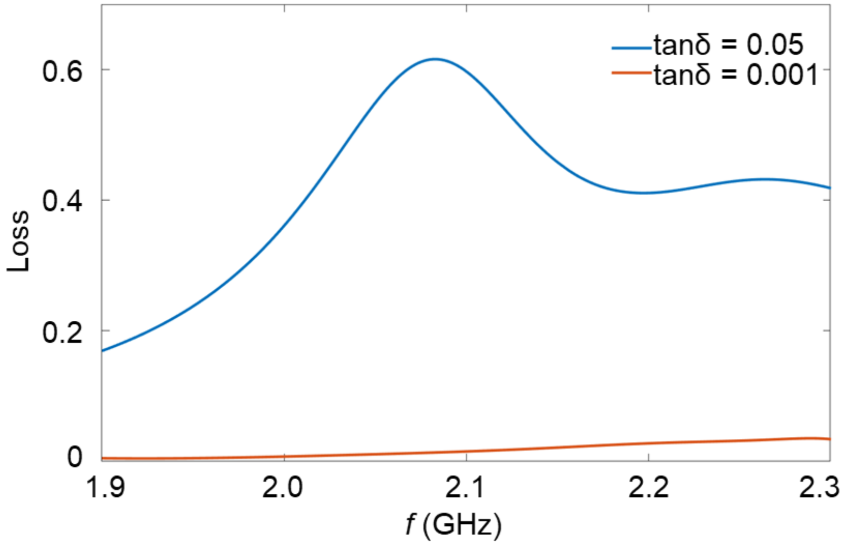

Fig. S10. Loss of the microwave metasurface unit under different substrate loss tangents. The maximum of increased loss is 0.6, comparable to the loss typically observed in optical metasurfaces.

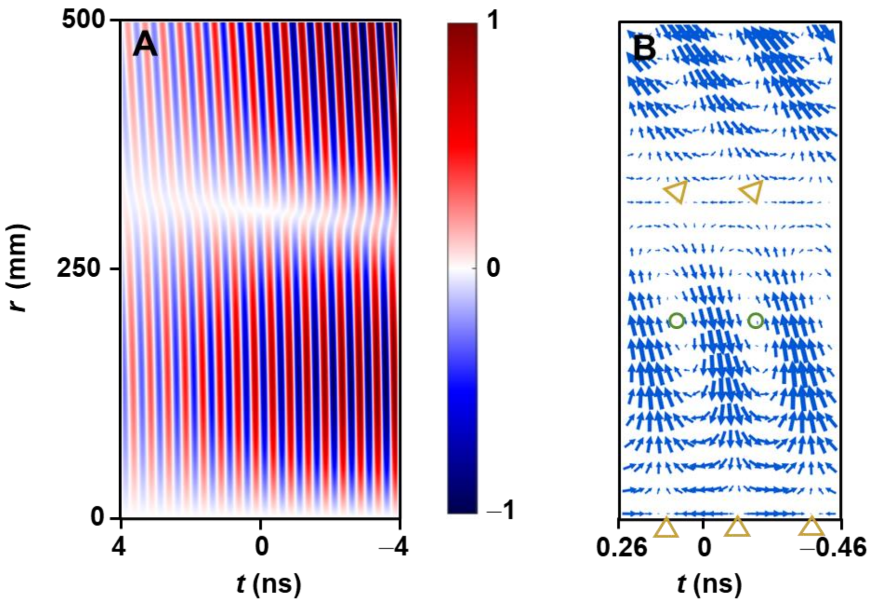

Fig. S11. Field distribution on a radial cross-section when the substrate of the metasurface has a loss tangent of 0.05. (**A**) Radially polarized electric field distribution, (**B**) vector field distribution. In (**A**), the color represents field intensity; in (**B**), the arrow, circle and triangle represent electric field vector, vortex ring and saddle point, respectively.
